# Diagnosis of Systemic Lupus Erythematosus in a Polynesian Male with a History of Rheumatic Fever: A Case Report and Literature Review

**DOI:** 10.1155/2018/8762482

**Published:** 2018-12-30

**Authors:** Charyse Diaz, Matthew A. Lim, Chloe A. Liu, Chloe S. Miwa, Darcy Tokunaga, Faith D. Hamamura, Kara Yamamoto, David Kurahara

**Affiliations:** Department of Pediatrics, University of Hawaii, John A. Burns School of Medicine, Kapi'olani Medical Center for Women and Children, Honolulu, HI 96826, USA

## Abstract

The presence of rheumatic heart disease (RHD) and systemic lupus erythematosus (SLE) has rarely been described in one patient. This report describes an adolescent Polynesian male with RHD who developed SLE years later. Initially, he fulfilled modified Jones criteria for rheumatic fever with aortic insufficiency, transient arthritis, elevated streptococcal titers, and a high erythrocyte sedimentation rate with a negative antinuclear antibody (ANA). He responded well to nonsteroidal anti-inflammatory and penicillin prophylaxis, which supported the diagnosis of rheumatic fever. Five years after his RHD diagnosis, he developed pancreatitis with glomerulonephritis, nephrosis, and pancytopenia. In addition, laboratory results revealed that he had multiple autoantibodies: anti-Sm and extremely elevated anti-dsDNA and ANA, fulfilling diagnostic criteria for SLE. The patient was treated, and he responded to pulse steroids followed by oral steroid therapy. To our knowledge, there are no known reported cases of a patient who was diagnosed with both RHD and SLE and met the clinical criteria for both diseases. The rarity of this concurrent disease process in one patient suggests a possible overlap in humoral immunity toward self-antigens as well as ethnic variability that increases predisposition to rheumatologic diseases.

## 1. Introduction

Acute rheumatic fever (ARF) and rheumatic heart disease (RHD) is a disease process still endemic and predominant in the Pacific as reflected by having the highest per capita incidences of ARF in the world [1]. The prevalence of systemic lupus erythematosus (SLE) and ARF in this region has been studied, but there is limited literature regarding a clinical correlation between the two aforementioned rheumatologic diseases [2]. To our knowledge, this article represents the first reported case of a male patient with RHD who subsequently developed SLE, suggesting a possible B-cell humoral abnormality.

## 2. Case Report

A 16-year-old Samoan male with a history of RHD, diagnosed in 2013, subsequently developed SLE in 2018. His initial presentation of RHD included mild aortic insufficiency, arthritis to his fifth metatarsal, positive streptozyme test, anti-DNase B 789 U/ml (normal < 170), ANA < 40, negative rheumatoid factor, and sedimentation rate up to 93 mm/hr. Two days after admission, he had a C-reactive protein 75.9 mg/L (normal < 1). Once his rheumatic fever resolved, he was placed on monthly parenteral benzathine penicillin G prophylaxis (BPG) for which he had incomplete compliance. He remained asymptomatic until he developed SLE five years later.

In January 2018, he developed fever, oral ulcers, pancreatitis, elevated ANA titers, elevated anti-Sm, elevated dsDNA, pancytopenia, and proteinuria. He initially presented after having a fever, coryza, and cough with a sore and hoarse throat. He then developed cold sores on his lips and roof of his mouth. After several days, the cough, runny nose, and fever resolved, but his sore throat persisted with subsequent lip swelling and redness accompanied with painful ulcers on lips and palate. Throughout this period, his solid food intake decreased and he reported a 30-pound weight loss.

He was evaluated in the outpatient setting after developing diffuse abdominal pain without fever, nausea, vomiting, or jaundice. The pain gradually became more constant and localized to the midepigastric and right upper quadrant of his abdomen with a noted blood pressure (BP) of 60/40, but no interventions were reported by his mother. He returned and saw his primary care physician who noted a BP of 84/52. Thus, he was transported to the hospital by ambulance, given a fluid bolus, and admitted for hypotension for potential sepsis and further workup.

His only previous significant medical history, aside from RHD, was stage 1 hypertension resolved at his last cardiology follow-up. Reviewing his outpatient records revealed sporadic BPG administration.

A general physical exam was significant for a fever of 38.3°C, ulcers with erythema on the hard palate, left lower, and upper lip, and oropharynx with petechial spots. Additionally, there was a blanching scaly rash noted to his helices and postauricular areas of ears with extension to the adjacent scalp on the left side. Although his overall abdomen was soft without organomegaly, he possessed tenderness to palpation of his upper quadrants. The lymphadenopathy of his superior left posterior cervical chain was unusual with a small 1-2 centimeter lymph node tender to palpation as well as a small, mobile, nontender lymph node of his left axilla. Further physical exam was otherwise normal.

Initial laboratory findings upon admission were significant for pancreatitis with a lipase of 3,140 U/L (normal < 58), transaminitis (AST 367 U/L and ALT 166 U/L) with elevated PTT, and pancytopenia with mild normocytic anemia, leukopenia, and thrombocytopenia. Bone marrow biopsy was normal after an elevated ferritin level of 2,159 mg/ml (reference range 36–311 ng/mL) was found. Multiple triglyceride and fibrinogen levels were found to be within the normal range which made hemophagocytosis less likely. Urinalysis results revealed proteinuria and trace leukocyte esterase. Initial infectious workup was unremarkable with negative antibodies toward viruses known to cause hepatitis. However, initial imaging on the ultrasound showed mild bilateral kidney enlargement but was otherwise unremarkable.

He was started on piperacillin-tazobactam for a four-day course due to progressive pancytopenia. His serum returned positive for salmonella, typhi, and paratyphi antibodies, so he was started on daily ceftriaxone until gastrointestinal PCR returned negative for salmonella. Rheumatologic workup was as follows: ANA of 1,280, anti-dsDNA of 20,480 (normal < 10), and anti-Sm antibodies elevated at >8. He completed four days of pulse steroids before initiation of steroid taper regimen. Nephrology followed him for lupus nephritis with proteinuria and an initial random protein-creatinine ratio at 610. Despite initiation of steroids, proteinuria persisted. For his pancreatitis, he was first placed on a clear diet, which he did not tolerate, and the diet was changed to nothing by mouth. He was started on partial parenteral nutrition and eventually total parenteral nutrition until he could tolerate diet. Finally, he received his monthly BPG prior to discharge.

## 3. Discussion

To our knowledge, this is the first presented case of a male patient developing RHD and later developing SLE as two separate diagnoses. This case may suggest a clinical correlation between two rheumatologic diseases. It is unlikely that these two relatively rare diagnoses could have occurred in one individual due to chance but this is a possibility. Our group had studied the point prevalence of SLE in this state and found a point prevalence in the urban areas of 24.0 per 100,000 [3], making the diagnosis of SLE less likely due to coincidence. However, in another study looking at ethnic groups at risk for rheumatic disease, we found that Samoan children in our rheumatic population had odds ratios of developing ARF of 120.7 and SLE of 11.5 [2]. During that time period of 6 years, there were no Samoan children who developed juvenile rheumatoid arthritis, so it was felt this ethnic group was at greater risk of developing autoantibody-producing disease over cell-mediated disease. This ethnic group at higher risk for this type of autoimmune disease warrants further study.

A review of the literature revealed one case of a female patient clinically diagnosed with RHD who was later diagnosed with SLE 22 years after her initial diagnosis [4]. However, our case had more data including laboratory values that made SLE less likely during the initial diagnosis of RHD. The only other similar case in our literature review was that of a 15-year-old female, who was initially misdiagnosed with Sydenham's chorea associated with ARF, but was later found to have lupus after careful follow-up [5]. These referenced cases focused on female patients. It is important to emphasize that the incidence of lupus in males is lower than females, so to have two rheumatologic disorders in one male patient should be even rarer [6]. This may point to an abnormality in humoral immunity and self-tolerance. This case is important to the literature because a diagnosis of SLE could have been missed if symptoms were solely attributed to the recurrence of RHD. Therefore, it was important to approach each presentation of symptoms with a broad differential of potential rheumatic diseases to make the correct diagnosis.

Although there are no supporting evidence about the related pathogenesis between SLE and RHD, there are some parallels between rheumatic fever and antiphospholipid antibody syndrome, specifically in pathogenesis and overlap in humoral immunity toward self-antigens like human cardiac myosin [7]. Sera from SLE, SS, and poststreptococcal acute glomerulonephritis patients demonstrated idiotypic reactivity with anti-My1 in one study [8]. Affinity-purified anti-myosin antibodies from SLE, SS, and ARF sera also reacted strongly with anti-My1, indicating that immunoglobulins produced in these diseases share idiotypic determinants. The data demonstrated an association of one myosin idiotype with poststreptococcal sequelae like ARF and SLE, which is similarly seen in our patient. Further research would be needed to investigate this particular immune response in patients with two rheumatic illnesses such as ours.

Additionally, pancreatitis may be underrecognized in patients newly diagnosed with SLE since both pancreatitis and SLE are uncommon in children [9]. The incidence of lupus pancreatitis is between 0.4 and 1.1 cases per 1000 lupus cases per year [10]. Lupus pancreatitis is thought to be caused by global multisystemic inflammation, such as vasculitis, immune complex deposition, microthrombi, and vascular intimal thickening, and ischemia [11]. T-cell infiltration and complement activation are also possible causes of pancreatitis seen in SLE [12]. However, the rarity of lupus pancreatitis suggests that chronic inflammation is not the only precipitating factor of this disease. The more common causes of pancreatitis include hepatobiliary pathology, alcohol, hypertriglyceridemia, and medications. Our patient did not present with any of these common causes of pancreatitis, and improvement with corticosteroid therapy points to the multisystemic inflammation in SLE as the etiology of his pancreatic inflammation. It was important in this case to have a high clinical suspicion for this complication and to test for lipase levels was critical for a good outcome in his disease management, as workup and management was directed at this potentially life-threatening complication of SLE.

Another consideration in this case was possible salmonella-triggered macrophage activation syndrome (MAS). MAS is a condition with an excessive activation and proliferation of T lymphocytes and macrophages with massive hypersecretion of proinflammatory cytokines and can occur in juvenile idiopathic arthritis and less often SLE [13]. With an elevated ferritin level, this was considered and a negative bone marrow biopsy for hemophagocytosis along with normal triglyceride and fibrinogen levels helped to rule this out. Ferritin is an acute phase reactant, and we have seen them quite high in our rheumatic population, and considering MAS with hyperferritenemia has become much more commonplace in our practice here.

## 4. Conclusion

We describe a patient with a history of RHD who years later developed SLE with pancreatitis. This diagnosis suggests a possible overlap between rheumatic fever and SLE in pathogenesis or that his particular ethnic group is at higher risk for autoantibody-mediated disease. These possibilities warrant further investigation.
